# Assessing User Retention of a Mobile App: Survival Analysis

**DOI:** 10.2196/16309

**Published:** 2020-11-26

**Authors:** Yu-Hsuan Lin, Si-Yu Chen, Pei-Hsuan Lin, An-Shun Tai, Yuan-Chien Pan, Chang-En Hsieh, Sheng-Hsuan Lin

**Affiliations:** 1 Institute of Population Health Sciences National Health Research Institutes Miaoli Taiwan; 2 Department of Psychiatry National Taiwan University Hospital Taipei Taiwan; 3 Department of Psychiatry College of Medicine National Taiwan University Taipei Taiwan; 4 Institute of Health Behaviors and Community Sciences College of Public Health National Taiwan University Taipei Taiwan; 5 Institute of Statistics National Chiao Tung University Hsinchu Taiwan

**Keywords:** smartphone, passive data, user retention, mobile application, app, survival analysis, work hours

## Abstract

**Background:**

A mobile app generates passive data, such as GPS data traces, without any direct involvement from the user. These passive data have transformed the manner of traditional assessments that require active participation from the user. Passive data collection is one of the most important core techniques for mobile health development because it may promote user retention, which is a unique characteristic of a software medical device.

**Objective:**

The primary aim of this study was to quantify user retention for the “Staff Hours” app using survival analysis. The secondary aim was to compare user retention between passive data and active data, as well as factors associated with the survival rates of user retention.

**Methods:**

We developed an app called “Staff Hours” to automatically calculate users’ work hours through GPS data (passive data). “Staff Hours” not only continuously collects these passive data but also sends an 11-item mental health survey to users monthly (active data). We applied survival analysis to compare user retention in the collection of passive and active data among 342 office workers from the “Staff Hours” database. We also compared user retention on Android and iOS platforms and examined the moderators of user retention.

**Results:**

A total of 342 volunteers (224 men; mean age 33.8 years, SD 7.0 years) were included in this study. Passive data had higher user retention than active data (*P*=.011). In addition, user retention for passive data collected via Android devices was higher than that for iOS devices (*P*=.015). Trainee physicians had higher user retention for the collection of active data than trainees from other occupations, whereas no significant differences between these two groups were observed for the collection of passive data (*P*=.700).

**Conclusions:**

Our findings demonstrated that passive data collected via Android devices had the best user retention for this app that records GPS-based work hours.

## Introduction

In the past decade, smartphones have become nearly ubiquitous. Over 3 billion smartphones have internet subscriptions, and each device has the information processing capacity of supercomputers of the 1990s [1]. Even in areas without easy access to clean water, ownership of a smartphone and rapid access to information have become symbols of modernity [2]. Although most comparable sources of big data are scarce in the world’s poorest nations, mobile phones are a notable exception [3]. Individuals’ history of mobile phone use can be used to infer their socioeconomic status [4]. Mobile apps could also help to fill the gap of health inequality, such as that for workers with extremely long work hours.

User retention is defined as the number of initial users who are still active in a given time frame. User retention is usually calculated as the number of those who are still active divided by the total number of users registered [5]. User retention enables designers to understand how often the users return to use the available service of a mobile app. Tracking user retention is both important and difficult, because the majority of mobile app users only use apps over a short timeframe [6] and report that the most important and acceptable components of a mobile app are ease of use and time, with the average time of use being 9.3 seconds. Gathering enough data to make reliable inferences of the retention rate over long timespans is also a challenge. Recently, user retention was adopted as a crucial index to evaluate the effect and utility of mobile apps designed for self-management. Previous studies have shown that users benefit from long-term engagement with an app [7]. However, the lack of studies with large samples using a mobile app may signal a need for additional studies on the potential use of a mobile app to assist individuals in changing self-management behaviors, such as health behaviors [5].

There were over 90 million documented mental health app installations by the end of 2018 [8]. Although mobile apps have been considered a feasible and acceptable means of administering health intervention, most literature regarding health apps has focused on preventing and managing chronic disease, self-monitoring of health behaviors, or content analyses of health and fitness apps [5]. However, studies investigating the utility of mobile apps as a health intervention were mostly executed in empirical study settings. Understanding patterns of real-world usage of health apps is key to maximizing their potential to increase self-management of care by the public [8]. Although the number of app installs and daily active minutes of use may seem high, only a small portion of users actually use apps for a long period of time [8]. A usage analysis of user engagement of unguided mental health apps found the general user retention is poor, with a median 15-day retention of 3.9% and 30-day retention of 3.3% [8]. Therefore, how to enhance long-term engagement of real-word usage of health apps may be crucial for both designers and users.

Passive data are defined as data that are generated without any direct involvement from the subject, such as GPS traces and phone call logs. By contrast, active data are defined as data that require active participation from the subject, such as surveys and audio samples [9]. Passive data generated by mobile apps have transformed the manner of traditional assessments based on active data. In 2003, the Accreditation Council for Graduate Medical Education implemented work hour limits for all physicians in training in the United States. However, surveying medical interns’ compliance with these work hour limits using a traditional assessment took 2 years, and the national survey was published in 2006 [10]. In addition, these self-reports could not reflect fluctuations in work hours in a timely manner, especially for medical staff with frequent on-call duties. Nowadays, smartphones offer us objective and ecological sources of measurement that continuously and passively collect data. These reliable, quantitative data could facilitate real-time policy evaluation and target resources to those with the greatest requirement, even in remote and inaccessible regions. In addition, policy regarding resident physician work hours has shifted frequently in recent years [11,12]; assessing user retention of an app like “Staff Hours” is useful to track the implementation of a work hours policy, as well as the resident physicians’ compliance with work hour limits.

We developed an iOS and Android smartphone app called “Staff Hours” that automatically calculates users’ work hours through GPS data. This passive data collection by “Staff Hours” is similar to that of our previous apps, “Know Addiction” and “Rhythm,” which collect and calculate smartphone screen time and sleep time, respectively [13-18]. However, these two apps were experimentally used for months, and user retention of an app collecting passive data in a natural setting is still unknown. The inherently dynamic nature of apps adds to the challenge of developing reliable metrics of app users’ retention. In addition, mobile app development is largely consumer-led and commercial-driven, and the evaluation of user retention is often app-centered and not user-centered. For example, a study tracking the longitudinal availability of mental health apps reported that these apps have a half-life; after a certain amount of time, an app may no longer be available for public use [19]. These app-centered metrics may have a low correlation with the apps’ clinical utility or usability [20].

Despite the ability of passive data collection by smartphone apps, little is known about user retention in the real world. Of particular interest are potential factors associated with the user retention of mobile apps. We hypothesized that user retention for passive data collection is higher than that for active data collection. We further hypothesized that power-saving operating systems and the target audience increase user retention for passive data and active data, respectively. The primary aim of this study was to quantify the user retention for the “Staff Hours” app using survival analysis. The secondary aim was to compare user retention between passive data and active data, as well as factors associated with the survival rates of user retention.

## Methods

### Participants

We collected data from 421 office workers from August 2018 to March 2019 from the “Staff Hours” database, which is owned by the National Health Research Institutes. This newly developed “Staff Hours” app automatically estimates users’ work hours daily through a GPS record using an algorithm. All participants were volunteers who were interested in their work hours and had successfully installed this app. We defined any data uploaded to our server during the first 28 days of their registration as successful installation of this app. We excluded 79 of the 421 app users who did not provide demographic data such as gender and age. Otherwise, app users had to provide their demographic data and occupation. A total of 342 participants (224 men; mean age 33.76 years, SD 7.01 years, range 20-59 years) were included in this study. Most of the participants (286/342, 83.6%) were medical staff, and most of the medical staff (128/286, 44.8%) were trainee physicians (resident physicians). The “Staff Hours” app is only available in Taiwan, and consent was obtained from all users to allow their data to be collected electronically before installation. Different versions of this app were available on the Android or iOS platform. The study was approved by the Institutional Review Board of the National Health Research Institutes. All clinical investigations were conducted according to the principles expressed in the Declaration of Helsinki.

### Measures

#### Designing the App — Staff Hours

**Figure 1:**
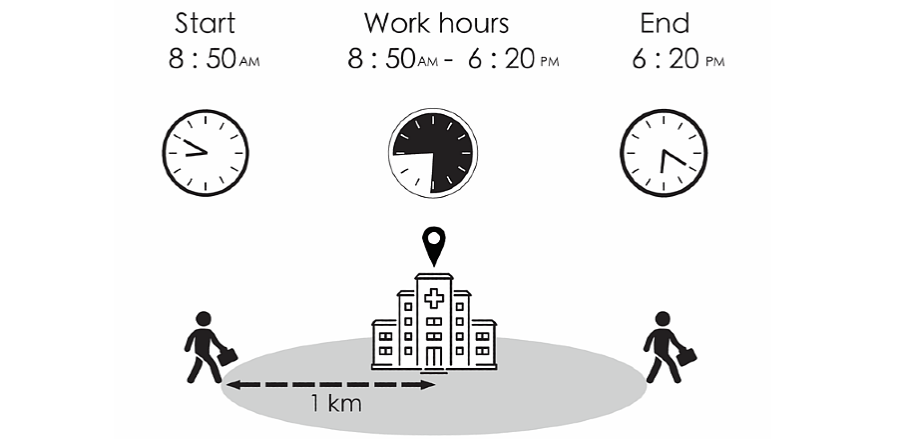
Recording process of GPS-defined work hours. In this example, the user walks into and leaves the 1-km radius centered on the workplace at 8:50 AM and 6:20 PM, respectively. The app then automatically generates GPS-defined work hours in real time (8:50-6:20).

**Figure 2:**
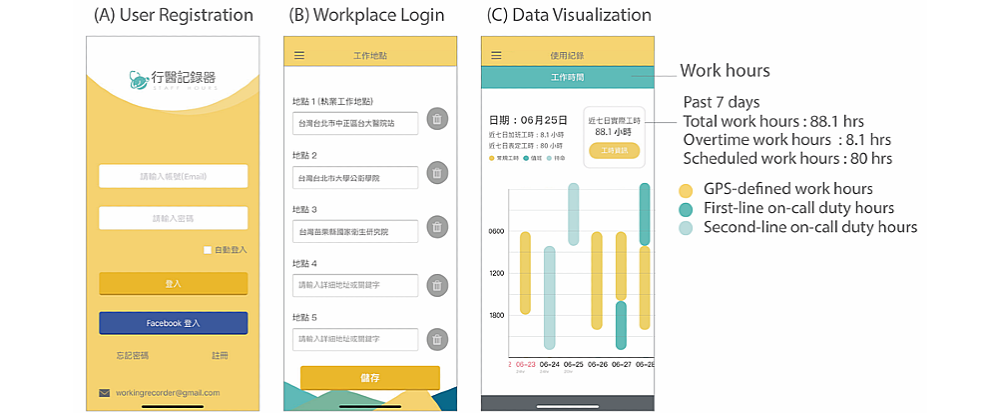
Screenshots of the "Staff Hours" app: (A) home screen and registration; (B) workplace login; (C) data visualization.

The “Staff Hours” app is designed to automatically record GPS data in the background without interrupting the smartphone operating system. In the beginning of installation, users have to provide at least one workplace location; the app can track up to 5 locations simultaneously. The location is transformed to longitude and latitude with the Google map format. The process of recording the GPS-defined work hours is illustrated in Figure 1. The GPS detection range is a 1-km radius around the workplace, and the recording of work hours starts when the workplace location is within range for a consecutive 30 minutes. Similarly, the recording of work hours ends when there is a consecutive 30-minute period without the workplace within 1 km of the device. To save battery power, the sampling rate of GPS data is fixed at 10 minutes. The user interface of the “Staff Hours” app is shown in Figure 2. In addition to GPS-defined work hours, users can set up their work schedules in this app. The user-inputted “scheduled work hours” include “regular work hours” and “on-call duty hours.” Aside from total work hours, the app also provides the output “overtime work hours” using the formula of “total work hours” minus “scheduled work hours.”

#### Collecting Passive Data — GPS-Defined Work Hours

Collecting and uploading data to the dedicated lab server occur automatically in the app background, and no interference with the daily routine of the smartphone use is observed. “Staff Hours” collects these GPS-defined work hours in the Android operating system, but the app stops during background refresh in the iOS. These GPS-defined work hours data are called “passive data” throughout this article since the data collection does not require any active participation from the smartphone user.

#### Collecting Active Data — Mental Health Survey

Every morning, there is a notification to remind the app users to examine their work hour records. On the first day of every month, this app pushes a notification for an 11-item mental health survey. This questionnaire includes the Patient Health Questionnaire (PHQ-9) for depressive symptoms [21], experience with phantom vibration and ringing syndrome [22], and one item for quality of life measured on a 4-point Likert scale. In contrast to passive data, self-reported data from the questionnaire are called “active data” throughout this article.

#### Defining User Retention

Considering the intermittent usage of this app and patterns of uploaded data, we herein define that user retention of this app stopped on the first date of a 28-day period without any data being uploaded. For every smartphone, user retention was measured separately for passive and active data.

### Statistical Analysis

In this study, the user retention length and indicator of whether an individual uninstalled the app were treated as a survival event. The Kaplan-Meier estimator, a standard nonparametric statistic used to estimate survival function of time-to-event data, was applied to measure the survival rate of user retention. A log-rank test was used to examine the difference in survival curves among different subpopulations or data collection modes (ie, active vs passive). A Cox proportional hazards model (Cox model) was used to assess the association between the hazard rate of user retention and two factors (smartphone users’ operating system and occupation). Occupations included resident physicians, visiting staff, medical students, nurses, and others. Comparing different occupations, we focused on resident physicians because their work hours are the longest among all occupations [22-27]. All analyses were performed using R software (version 3.4). The survival analysis was based on the package “survival” [28]. A *P* value less than .05 was considered statistically significant.

### Patient and Public Involvement

No patients nor public members were involved in our work.

## Results

**Figure 3:**
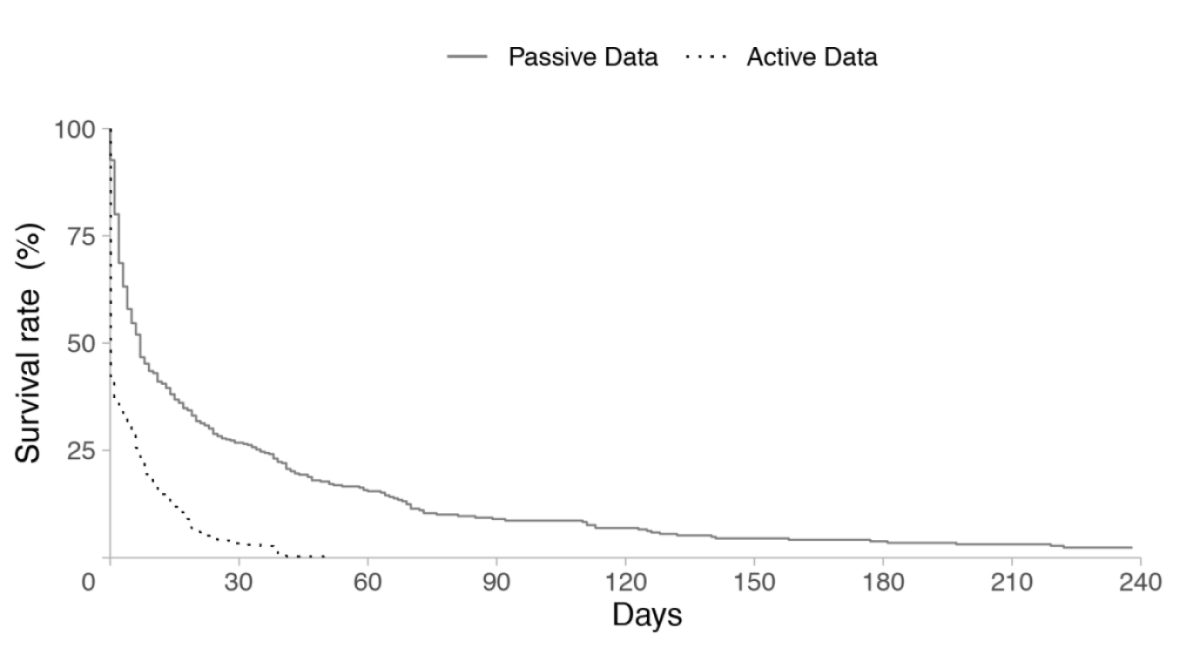
Survival curves of passive and active data.

We first compared user retention time between self-reported and GPS-defined work hours by analyzing active and passive data recorded using the “Staff Hours” app. Survival curves of user retention by passive and active data are illustrated in Figure 3. The survival rate of user retention for passive data was 46.7% after 1 week, whereas the rate for active data was 22.2%. After 1 month, the survival rate for passive data was 27.3%, and that for active data was 3.4%. The log-rank test results show that the survival time of passive data was significantly higher than that of active data (*P*=.011). This result is consistent after conditioning on the different operation systems, which is shown in Multimedia Appendix 1.

**Figure 4:**
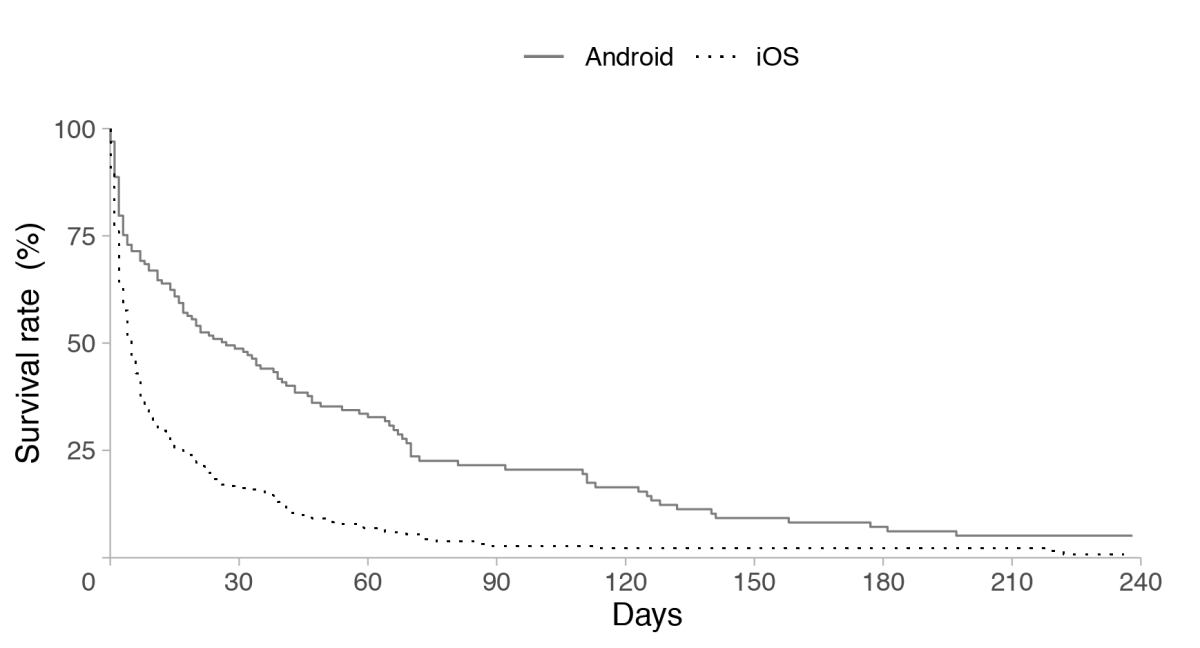
Survival curves of GPS-defined work hours between the operating systems.

**Figure 5:**
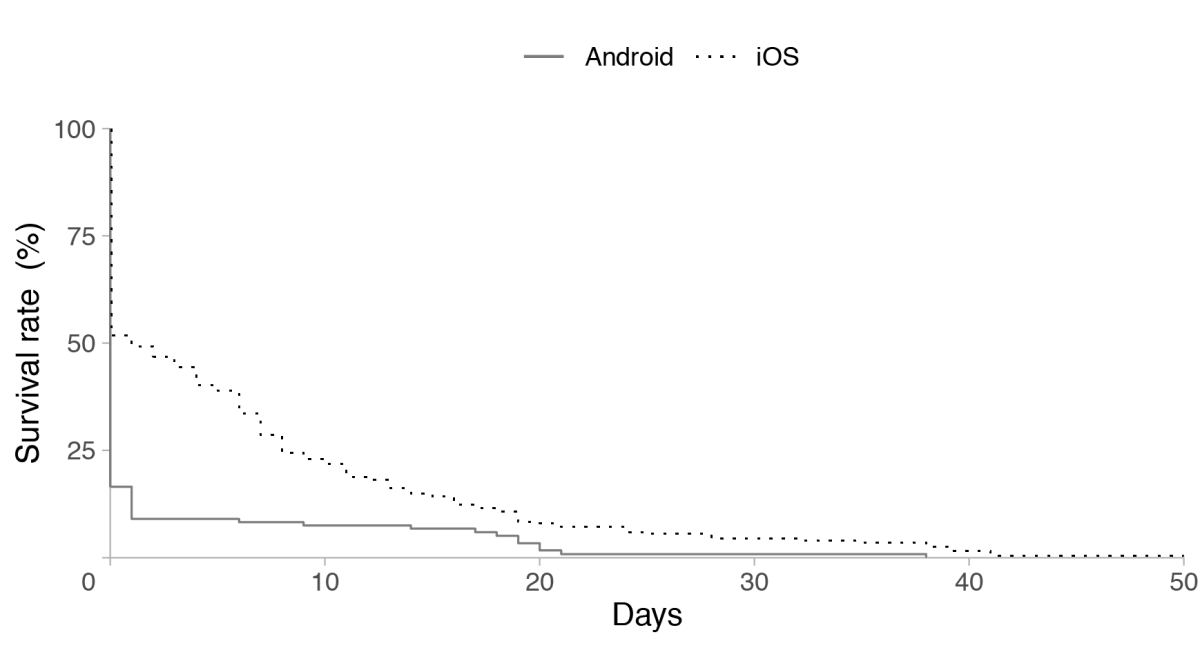
Survival curves of self-reports between the operating systems.

The survival curves for the 2 operating systems (Android and iOS) for passive data are shown in Figure 4 and for active data are shown in Figure 5. For passive data, 1-week survival rates of user retention for Android and iOS were 69.2% and 36.2%, respectively. After 1 month, the survival rate for Android reduced to 49.5%, whereas the rate for iOS reduced to 16.7%. For active data, 1-week survival rates of user retention for Android and iOS were 8.3% and 28.6%, respectively. After 1 month, the survival rate for Android reduced to 0.8%, and the survival rate for iOS reduced to 4.5%. The log-rank test revealed that patterns of user retention varied based on the operating system, with Android users maintaining a significantly higher survival rate of user retention for passive data across time (*P*=.015; Figure 4) than iOS users. By contrast, the patterns of user retention for active data showed no significant difference between Android and iOS (*P*=.700; Figure 5).

The results of the Cox model are shown in Table 1 and Multimedia Appendix 2. User retention for the collection of passive data via Android devices was higher than that for iOS. Resident physicians had higher user retention for the collection of active data than those with other occupations (*P*=.041), whereas no significant differences were observed between these two groups in passive data collection.

**Table 1:** Cox proportional hazards model.

Comparison	Passive data		Active data	
	Hazard ratio (95% CI)	*P* value	Hazard ratio (95% CI)	*P* value
Operating system (iOS vs Android)	2.688 (1.846-3.913)	.012	1.707 (0.625-4.661)	.610
Occupation (resident physicians vs others)	0.711 (0.497-1.017)	.361	0.119 (0.044-0.322)	.041

## Discussion

### Principal Findings

Our results suggest that quantifying user retention of an app using survival analysis is feasible. The survival curves of user retention for passive and active data collection provide a reference for user retention of an app that was not actively promoted. This user retention obtained via survival analysis could be a useful indicator to monitor the effects of advertising campaigns or promotional activities for any app. Our data help to study the critical period of user retention. The survival rate of 46.7% for the collection of passive data was until the first week, and this suggests that more than half of the app users quit using the app or technical problems interrupted the passive data collection upon app installation. Helping first-time users to navigate this app and delivering frequently asked questions through a push notification during this critical period could be valuable, and this survival rate implies potential technical problems in the collection of passive data via this app. Previous studies have also demonstrated similar results — that user retention significantly decreases during the first week [29]. Among those who downloaded the “PTSD Coach” app (n=153,834), designed for American veterans to reduce post-traumatic stress disorder symptoms, 61.1% returned to use the app after the first day of installation. Over half of users (52.1%) continued to use the app or used it at least one time beyond the first week of download. A commonly observed pattern is the user downloading an app, quickly scanning it, and determining personal relevance. For users who may only open the app a single time, it is the only opportunity to capture their interest. Therefore, mobile health app developers have also suggested that health care professionals “need to very carefully manage the initial phases of somebody using this kind of technology and make sure they’re well monitored” [30].

“Staff Hours” provides the first model to examine user retention for passive and active data collection simultaneously. Although a higher user retention for passive data collection over active data collection seems intuitive, we first quantified the survival rate of user retention and examined the factors associated with this survival rate. In addition, we established a user-centered evaluation of the mobile app’s user retention based on an app-centered longevity evaluation [19]. The present analysis may be a useful metric of usability evaluation for a mobile health app, even a software medical device. Apart from the efficacy and safety evaluation in the traditional registration of new drugs or medical devices, the American Psychiatric Association proposed an app evaluation framework that emphasizes usability or engagement [31]. The engagement evaluation includes how many patients became stuck when using an app or found them difficult to use. This user-centered evaluation encourages app developers to involve patients or potential target users in the development of their health apps.

Our findings show that user retention for the collection of passive data via Android devices was higher than that with iOS. Platform-related differences in user retention resulted from the differences in the GPS data collection between Android and iOS. Specifically, “Staff Hours” will stop collecting data during a background refresh in iOS but not in Android. The fact that passive data collection consumed more electricity in iOS could explain the lower user retention in iOS for passive data. By contrast, no significant platform-related differences in user retention in the collection of active data were observed. Because active data collection differs from passive data collection, which was generated continuously from day-to-day human-machine interaction, user retention is not associated with operating systems. In summary, platform-related differences in user retention in the collection of passive data, but not of active data, imply that electricity consumption may be of a particular concern to smartphone users, given the challenges of developing apps for 2 distinct platforms.

Our results provide novel information regarding resident physicians’ user retention in the collection of active data. This higher user retention suggests that resident physicians represent the right target audience for this app. “Staff Hours” was designed not only to automatically record work hours but also to survey staff’s mental health during moments of long work hours. Resident physicians had extremely long work hours and mental health issues at the time. As a result, they were more motivated to continuously interact with this app. The PHQ-9 for depressive symptoms, phantom vibration, and ringing syndrome in this mental health survey is specifically designed for staff with excessive work hours such as resident physicians. A systematic review and meta-analysis showed a high prevalence rate of 28.8% of depression or depressive symptoms among resident physicians [32]. Our previous study also showed that more than 85% of medical interns with 86.7 work hours per week experienced phantom vibration and ringing syndrome, and these syndromes significantly reduced 2 weeks after their internship [27]. Such mental health surveys on smartphones are important because smartphone use may reduce bias in the form of the Hawthorne effect. This effect has been reported in a previous study to demonstrate that PHQ-9 depressive symptom scores recorded from the app were more sensitive in detecting suicidal behavior than the traditionally administered PHQ-9 [33]. Mental health and work hours are both important for resident physicians, but excessive work hours did not represent poor mental health. Our recent study showed that medical interns with an additional 10 work hours per week (ie, average additional 2 work hours per day) had a relatively small increase in depressive symptoms, with a PHQ-9 score of 0.13 [34].

### Limitations

Several methodologic limitations should be noted when interpreting our findings. First, the rapid decrease in user retention in the collection of active data may be attributed to our definition of user retention, which stopped when no data were uploaded for more than 28 consecutive days. Furthermore, passive data and active data are collected from different sources (ie, GPS and self-report, respectively). Therefore, the difference in the user retention time is somehow affected by the characteristics of these resources. For example, sampling rates of active and passive data are unequal, and passive data are generated every 10 minutes, but active data, according to a mental health survey, are generated every month. Second, this definition of retention did not include users’ return to using the app after quitting for more than 28 consecutive days. Third, our sample size was not large enough to identify more factors associated with user retention and lacked significant power. In addition, all of the individuals only had one smartphone, so it was not feasible to compare the difference in retention time between iOS and Android operation systems for the same individuals. Finally, the user retention might also be different in an app that is more attractive than “Staff Hours.” In addition, the longer retention with our app on Android devices, compared with iOS devices, can be explained by the difference in electronic consumption, so the results might not be generalizable to other apps.

### Conclusions

In conclusion, we demonstrated that passive data collected via Android devices had the best user retention with our app that records GPS-based work hours. As a pilot study in this field, our results provide new insights into quantifying the usability of a mobile app using survival analysis. We also determined that the first week upon installation is the critical period for the app’s longevity. Analysis of user retention with additional apps is required to validate our methods.

## Appendix

Multimedia Appendix 1 Survival curves of GPS-defined work hours (passive data) vs self-reported work hours (active data) for each operating system.

Multimedia Appendix 2 Cox proportional hazards model adjusted by age and gender.

## Abbreviations

PHQ-9: Patient Health Questionnaire
