# Supplementary material for: Assessing User Retention of a Mobile App: Survival Analysis
Source: JMIR Mhealth Uhealth. 2020 Nov 26;8(11):e16309. doi: 10.2196/16309 (PMC7728530; doi:10.2196/16309)
Supplement: Multimedia Appendix 1 [file mhealth_v8i11e16309_app1.docx]

**Appendix for “Survival Analysis for Assessing User Retention of a Mobile Application”**

Yu-Hsuan Lin, Si-Yu Chen, Pei-Hsuan Lin, An-Shun Tai, Yuan-Chien Pan, Chang-En Hsieh, and Sheng-Hsuan Lin*


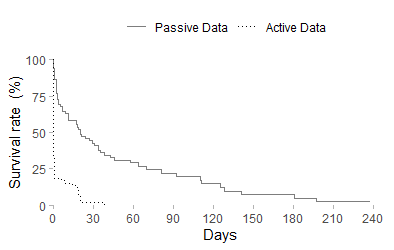


**Figure S1. Survival curves of GPS-defined work hours (passive data) v.s. self-report work hours (active data) under Android operating system.** The survival rate of user retention for passive data is significantly higher than that for active across time (p < 0.001).


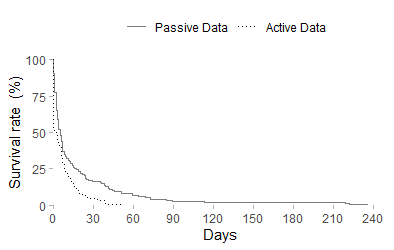


**Figure S2. Figure S1. Survival curves of GPS-defined work hours (passive data) v.s. self-report work hours (active data) under iOS operating system.** The survival rate of user retention for passive data is significantly higher than that for active across time (p < 0.001).
