# Supplementary material for: Assessing User Retention of a Mobile App: Survival Analysis
Source: JMIR Mhealth Uhealth. 2020 Nov 26;8(11):e16309. doi: 10.2196/16309 (PMC7728530; doi:10.2196/16309)
Supplement: Multimedia Appendix 2 [file mhealth_v8i11e16309_app2.docx]

**Table S1. Cox proportional hazards model adjusted by age and gender.**

|  | **Passive data** | |  | **Active data** | |
| --- | --- | --- | --- | --- | --- |
|  | Hazard ratio (95% CI) | p-value |  | Hazard ratio (95% CI) | p-value |
| Operation system (iOS vs. Android) | 2.420 (1.083, 5.408) | 0.031 |  | 1.079 (0.125, 9.287) | 0.945 |
| Occupation (resident physicians vs. others) | 0.646 (0.292, 1.426) | 0.279 |  | 0.115 (0.015, 0.903) | 0.040 |
